# Using the 4 pillars™ practice transformation program to increase adult influenza vaccination and reduce missed opportunities in a randomized cluster trial

**DOI:** 10.1186/s12879-016-1940-1

**Published:** 2016-11-03

**Authors:** Chyongchiou J. Lin, Mary Patricia Nowalk, Valory N. Pavlik, Anthony E. Brown, Song Zhang, Jonathan M. Raviotta, Krissy K. Moehling, Mary Hawk, Edmund M. Ricci, Donald B. Middleton, Suchita Patel, Jeannette E. South-Paul, Richard K. Zimmerman

**Affiliations:** 1 Department of Family Medicine, University of Pittsburgh School of Medicine, 4420 Bayard Road, Suite 520, Pittsburgh, PA 15260 USA; 2 Department of Family and Community Medicine, Baylor College of Medicine, Houston, TX USA; 3 Department of Behavioral and Community Health Sciences (MH, EMR), University of Pittsburgh Graduate School of Public Health, 130 DeSoto Street, Pittsburgh, PA 15261 USA; 4 Centers for Disease Control and Prevention (SP), Atlanta, GA USA; 5 Current address: Houston Methodist Primary Care Group, Houston, TX USA

**Keywords:** Influenza vaccine, Immunization, Adults

## Abstract

**Background:**

An evidence-based, step-by-step guide, the 4 Pillars™ Practice Transformation Program, was the foundation of an intervention to increase adult immunizations in primary care and was tested in a randomized controlled cluster trial. The purpose of this study is to report changes in influenza immunization rates and on factors related to receipt of influenza vaccine.

**Methods:**

Twenty five primary care practices were recruited in 2013, stratified by city (Houston, Pittsburgh), location (rural, urban, suburban) and type (family medicine, internal medicine), and randomized to the intervention (*n* = 13) or control (*n* = 12) in Year 1 (2013-14). A follow-up intervention occurred in Year 2 (2014-15). Demographic and vaccination data were derived from de-identified electronic medical record extractions.

**Results:**

A cohort of 70,549 adults seen in their respective practices (*n* = 24 with 1 drop out) at least once each year was followed. Baseline mean age was 55.1 years, 35 % were men, 21 % were non-white and 35 % were Hispanic. After one year, both intervention and control arms significantly (*P* < 0.001) increased influenza vaccination, with average increases of 2.7 to 6.5 percentage points. In regression analyses, likelihood of influenza vaccination was significantly higher in sites with lower percentages of patients with missed opportunities (*P* < 0.001) and, after adjusting for missed opportunities, the intervention further improved vaccination rates in Houston (lower baseline rates) but not Pittsburgh (higher baseline rates). In the follow-up intervention, the likelihood of vaccination increased for both intervention sites and those that reduced missed opportunities (*P* < 0.005).

**Conclusions:**

Reducing missed opportunities across the practice increases likelihood of influenza vaccination of adults. The 4 Pillars™ Practice Transformation Program provides strategies for reducing missed opportunities to vaccinate adults.

**Trial registration:**

This study was registered as a clinical trial on 03/20/2013 at ClinicalTrials.gov, Clinical Trial Registry Number: NCT01868334, with a date of enrollment of the first participant to the trial of April 1, 2013.

## Background

Adult influenza vaccination rates in the United States (U.S.) continue to languish at approximately 44 % as of 2014-2015 reported estimates [1], with annual increases hovering at less than 2 percentage points [2, 3]. The reasons for the disparity between reported rates in the community, the desired rate of 70 % [4] set forth in the Healthy People 2020, and meaningful annual increases are legion. They include logistical issues at the practice level such as storage, cost [5], return policies for unused vaccine, and choice of vaccines; strongly held personal beliefs about influenza vaccine at the patient level such as belief that the vaccine is not necessary, effective or safe [6]; and broader issues such as, changes in year-to-year effectiveness, timing of vaccine distribution [7] and vaccine administration reimbursement.

Several system-level efforts have been undertaken or implemented that have attenuated some of the barriers to vaccination. The Affordable Care Act requires that certain preventive services including immunizations be covered as part of basic care [8]. This mandate should effectively eliminate patients’ financial barriers to receiving influenza vaccine. The Centers for Disease Control and Prevention (CDC) encourages vaccine distribution policies that are designed to reduce regional and local shortages of vaccine, i.e., partial orders are shipped nationwide and orders are completed later as more stock becomes available [7]. An increasing number of hospitals and health systems now require their employees to receive influenza vaccine [9, 10].

To have any hope of attaining the Healthy People 2020 goal, a business-as-usual approach to increasing influenza vaccine uptake is no longer acceptable. A single strategy is unlikely to be successful. The Task Force on Community Preventive Services has recommended multi-strategy, evidence-based interventions [11] as an effective means of increasing immunization rates. These interventions should enhance access to vaccination services, increase community demand for vaccines, and improve provider- or system-based interventions.

The 4 Pillars™ Practice Transformation Program, also known as the 4 Pillars™ Immunization Toolkit, (4pillarstoolkit.pitt.edu) is a compilation of evidence-based best practices for increasing immunizations in primary care settings. It is built on decades of research by the investigators into the barriers to and facilitators of adult immunizations from the provider and patient perspectives, and trials of successful strategies. The 4 Pillars™ Program was the foundation of an intervention implemented in a randomized controlled cluster trial (RCCT), to increase adult immunization rates and reduce missed opportunities to vaccinate among patients of primary care practices in Pittsburgh and Houston [12]. The purpose of this study is to report on changes in adult influenza immunization rates and on factors related to the likelihood of receipt of influenza vaccine.

## Methods

This RCCT took place during 2013-2014 and 2014-2015 with baseline in 2012-2013, and was approved by the Institutional Review Boards of the University of Pittsburgh, Baylor College of Medicine and Harris Health System.

### Sample size and sites

Optimal Design software (University of Michigan, Version 1.77, 2006) was used to calculate sample size for a randomized trial seeking a 10-15 % absolute increase in vaccination rate with a minimum practice size of 100 patients. A sample size of 20 clusters or sites (10 Intervention and 10 Control practices) was determined to be necessary to achieve 80 % power with an alpha of 0.05. Eligible primary care family medicine (FM) and internal medicine (IM) practices from a practice-based research network (PBRN) in Pittsburgh (FM Pittnet), a clinical network in Southwestern Pennsylvania (UPMC Community Medicine, Inc.) and a PBRN in Houston (SPUR-Net) were solicited for participation. All Houston sites were publicly funded, safety net practices, caring for a disadvantaged population; whereas, Pittsburgh practices were smaller private practices or residency sites with patients from across the socioeconomic spectrum. When 25 sites agreed to participate, solicitation ceased. All sites used a common electronic medical record (EMR), EpicCare within their respective health systems.

### Cluster randomization

**Fig. 1:**
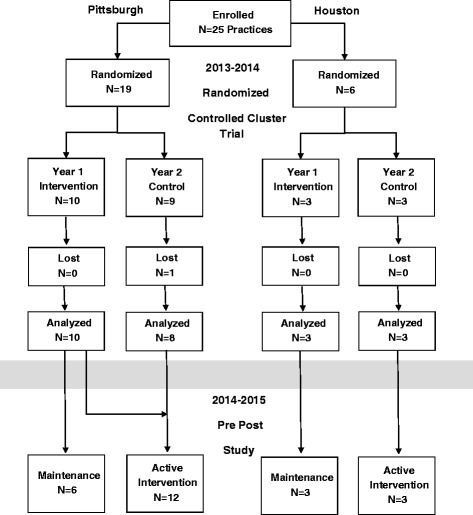
Randomization Scheme

Cluster randomization allocates clinical practices rather than individuals to the intervention arms [13]; thus, each site or office (some practices had more than one) was considered as a cluster. Eligibility requirements included having a significant adult practice, preliminary baseline vaccination rates for at least one adult vaccine <50 % and a willingness to make office changes to increase influenza, pneumococcal and Tdap vaccination rates. Participating practices were stratified first by metropolitan area (Pittsburgh or Houston), then in Pittsburgh only, by location (urban, suburban or rural) and by discipline (IM or FM). The practices were then randomized into the intervention or control arms within strata (Fig. 1). Year 2 control practices were informed that their intervention would take place the following season and were not contacted again until the next year.

### 4 Pillars™ program

The 4 Pillars™ Practice Transformation Program is founded on four evidence-based [11, 14, 15] key domains: Pillar 1 - Convenient vaccination services; Pillar 2 - Communication with patients about the importance of immunization and the availability of vaccines; Pillar 3 - Enhanced office systems to facilitate immunization; and Pillar 4 - Motivation through an office immunization champion (Champion). Table 1 describes the primary strategies contained in the 4 Pillars™ Program. The 4 Pillars™ Program includes background on the importance of protecting patients against vaccine-preventable diseases, barriers to vaccination from both provider and patient perspectives and strategies to eliminate those barriers. Practices were expected to implement strategies from each of the 4 pillars.

**Table 1:** Intervention strategies used to increase adult vaccination rates from the 4 pillars™ practice transformation program

Pillar 1	Convenient vaccination services
• Use every patient visit type as an opportunity to vaccinate.	
• Offer open access/walk-in vaccination during office hours.	
• Hold express vaccination clinics outside normal office hours where only influenza vaccine is offered and systems for check-in, screening, and record keeping are streamlined.	
• Create a dedicated vaccination station.	
• Extend the influenza vaccination season by vaccinating as soon as supplies arrive and continuing to vaccinate as long as flu is circulating in the community.	
Pillar 2	Communication with patients about the importance of vaccination and the availability of vaccines
• Train staff to discuss influenza vaccine during routine processes such as vital signs	
• Discuss the serious nature of influenza	
• Promote vaccination of staff to set a good example	
• Record telephone on-hold messages that advertise vaccine availability or promote vaccination.	
• Use posters/fliers/electronic message board/website postings/social media promoting vaccination	
• Conduct outreach by email, phone, text, mail, health portal, etc. that vaccines are due and/or available	
Pillar 3	Enhanced office systems to facilitate adult vaccination
• Assess vaccination eligibility for every scheduled patient at the beginning of the day and discuss in daily huddles	
• Assess immunizations as part of vital signs upon rooming patients and record outside vaccinations in EMR	
• Incorporate EMR prompts for vaccination into the workflow	
• Incorporate standing order programs (SOP) for vaccination by nurses and/or medical assistants into the workflow	
• Ensure sufficient vaccine inventory to handle increased immunizations	
• Promote simultaneous vaccination (e.g., offer other vaccines at the time of influenza vaccination)	
Pillar 4	Motivation through an office immunization champion
• Create a chart to track progress. Set an improvement goal and regularly track progress (e.g., daily or weekly). Post the graph of your progress in a prominent location and update it regularly.	
• Provide ongoing feedback to staff on vaccination progress using email, posted notices, making announcements, or using a combination of these. Encourage, nudge, and cheer as needed to keep up the momentum.	
• Report upon progress at staff or huddle meetings. Facilitate discussion at these meetings to identify which pillar activities are working, which are not working and why, and to identify changes that need to be made.	
• Create a competitive challenge among your staff for the most vaccinations given.	
• Provide rewards for successful results to create a fun-spirited environment that promotes vaccination across the practice. Ideas include: reward for highest vaccinator, team competitions, vaccination goal poster contest, etc.	

The 4 Pillars™ Program was provided as a printed and bound document, supplemented by a web-based practice transformation dashboard. The dashboard was developed from the work of Fixsen et al. [16], who established an empirically-based implementation framework that includes systematic uptake, establishment, and maintenance of research findings into routine practice. The core components include: staff selection and training on the specific evidence-based practices, expert consultation and coaching of staff and administration, program evaluation to assess and provide feedback, facilitative administrative supports to ensure data are used to focus and inform decision making, and systems interventions. Once the practice was registered, any staff member could log into the dashboard. The Champion was responsible for registering the practice and its staff members, and identifying strategies that the practice would implement. The 4 Pillars™ Program provided step-by-step guidance for implementing the strategies, and the dashboard showed the practices’ progress through the change process. Practices could monitor their progress on graphs that reported biweekly numbers of vaccines given.

### Interventions

The intervention was designed using Diffusion of Innovations theory [17], and included the 4 Pillars™ Practice Transformation Program, provider education, and one-on-one coaching of the immunization champion for each practice. Two of the investigators (AEB, MPN) visited the intervention sites each year to introduce the study and the 4 Pillars™ Program and to work with staff to develop practice-specific ideas for implementing strategies.

Each practice was asked to identify a Champion who would be responsible for updating the practice transformation dashboard as intervention strategies were employed to guide strategy implementation. Other tasks for the Champion included participating in the biweekly telephone call with a research liaison for coaching, ensuring that chosen strategies were being implemented, and working to maintain motivation of the staff.

Each practice was given a graph showing biweekly progress towards their goal based on an overall 20 % increase over the previous year’s total adult influenza vaccines given. These graphs were to be used by the Champion to encourage the staff to maintain motivation or stimulate additional changes to increase vaccination rates. The research liaison discussed these graphs with the Champions during their calls or visits.

### Data collection

De-identified demographic, office visit and vaccination data were derived from EMR data extractions performed by the UPMC Center for Assistance in Research using the eRecord and from a similar EMR extraction by staff of the SPUR-NET for the Houston sites. A longitudinal data base was created with only those patients who had a visit each year during all three years, creating a cohort of individuals who would have been patients of the practice during the entire study period.

### Statistical analyses

Descriptive analyses were performed for patient demographic characteristics (age, sex, race, ethnicity, and health insurance). Because of significant differences in patient populations, size and structure of the practices in Houston and Pittsburgh, the respective sites were grouped separately for analysis. Age was used as a continuous variable. Race and ethnicity were recorded differently in each city. In Pittsburgh sites, with few Hispanic patients, ethnicity was rarely recorded; hence patients were grouped by race into white and non-white with blacks and Hispanics assigned to the non-white group and only race data are presented and used in analysis. In Houston sites, with few non-Hispanic patients, race was rarely recorded; hence only ethnicity (Hispanic and non-Hispanic) is presented and used in analysis. Proportions were reported for categorical variables and means and standard deviations were reported for continuous variables.

The primary outcome measure, influenza vaccination rate was reported at the end of baseline (8/1/2012-1/31/2013) and the end of the intervention period (8/1/2013-1/31/2014) by site and by intervention group for the Year 1 RCCT analyses. At the end of Year 1, practices were offered the opportunity to continue active intervention during Year 2. Four practices opted to do so. At the same time, the Year 1 control sites began the intervention. For the Year 2 pre-post analyses, the four practices in Pittsburgh that continued the intervention in Year 2 were combined with the Year 1 control sites and were referred to as the active intervention group. The six practices that did not actively participate in Year 2 were referred to as the maintenance group. In Houston, the Year 1 intervention sites were referred to as the maintenance group and the Year 1 control sites that received the intervention in Year 2 were referred to as the active intervention group.

The August through January dates were used because the new seasonal influenza vaccines typically begin to arrive at practices in August and the majority of adult influenza vaccines are given by the end of January. Missed opportunities for all patients who were seen during the influenza vaccination seasons (8/1/2013-1/31/2014 and 8/1/2014-1/31/2015) were determined to be all visits in which an influenza vaccine was not received until vaccination or the end of the influenza season (1/31). The visit in which an influenza vaccine was given and post vaccination visits were not counted. Patients who were not seen at all during the influenza vaccination season were assigned a single missed opportunity to account for the fact that they should have been encouraged to come in for a vaccine. Missed opportunities were used as a measure of how well sites incorporated 4 Pillars™ Program strategies and how effective those strategies were. The proportion of patients with one or more missed opportunities was determined for each practice.

Cochran-Armitage trend tests were performed for determining percentage point differences between the baseline and intervention periods for the Year 1 RCCT and active intervention and maintenance for the Year 2 pre-post study for influenza vaccination rates and proportion of the patients with ≥1 missed opportunity [18]. Chi-square tests were used to compare the PP changes from baseline to follow up between groups.

To determine which factors were related to influenza vaccination rates, while accounting for the clustered nature of the data, generalized estimating equation (GEE) models were used to examine the likelihood of influenza vaccination after the intervention period, taking account of heterogeneity in demographic characteristics (including age, sex, and race/ethnicity and health insurance status) as well as the site - level variables intervention arm and proportion of patients with missed opportunities. An unstructured correlation matrix was used to accommodate the within-patient variation due to repeated annual measurement of influenza vaccination. Statistical significance of two-sided tests was set at type I error (alpha) equal to 0.05. All analytical procedures were performed using SAS® 9.3.

## Results

Twenty-four sites completed the Year 1 intervention (one site dropped out); their demographic and other characteristics are shown in Table 2. Houston sites were part of a publicly funded safety net system which were established to care for uninsured/underinsured patients; whereas, Pittsburgh sites were both training/residency sites and private practices. Houston sites were larger with higher proportions of Hispanic patients, female patients, and non-commercially insured patients than Pittsburgh sites. For these reasons, the results are presented separately for each city.

**Table 2:** Demographic and practice characteristics by practice and intervention group at baseline

Site	*N*	Age, yrs. Mean (SD)	Female, %	White, %	Non-white, %	Hispanic, %	Health insurance status		
							Medicaid,^a^%	Commercial, %	Medicare, %
Pittsburgh sites									
Intervention									
B	529	65.5 (14.6)	69.8	58.0	41.2	0.4	15.1	42.0	42.9
C	2179	60.1 (17.4)	60.3	99.4	0.3	0.1	11.7	58.5	29.8
D	3224	66.8 (14.7)	52.2	99.6	0.2	0.1	6.2	56.3	37.5
E	1392	56.5 (15.9)	58.6	95.1	4.7	0.1	14.5	61.9	23.6
G	417	67.0 (14.3)	52.0	82.5	16.6	0.0	5.3	49.2	45.6
H	306	66.7 (14.9)	59.2	62.4	37.0	0.0	13.7	41.5	44.8
F	3611	58.1 (17.0)	56.8	96.4	2.4	0.3	5.0	62.6	32.4
J	603	62.2 (18.6)	52.7	85.9	13.3	0.2	9.0	61.4	29.7
K	330	56.0 (17.7)	67.6	99.1	0.3	0.3	16.4	61.5	22.1
M	595	66.4 (14.9)	51.1	98.0	0.2	0.3	6.7	58.8	34.5
Total	13,186	61.7 (16.7)	56.7	94.3	5.0	0.2	10.0	59.4	30.6
Control									
N	2102	62.0 (16.4)	58.3	6.6	0.4	0.1	8.1	67.5	24.4
O	4324	57.2 (16.0)	53.9	98.6	0.7	0.1	7.4	65.0	27.6
R	2534	58.8 (14.6)	52.3	97.8	1.2	0.2	4.8	67.6	27.7
S	1645	43.6 (16.7)	75.1	53.3	45.7	0.8	58.4	23.4	18.2
U	2612	57.1 (17.3)	63.9	90.9	7.9	0.3	11.6	53.0	35.4
W	224	78.6 (10.4)	72.8	92.4	6.3	0.9	2.2	46.0	51.8
X	1010	53.3 (15.0)	46.6	96.5	2.0	0.0	12.0	64.5	23.6
Y	3334	60.2 (15.8)	58.9	97.6	1.7	0.1	7.9	60.7	31.5
Total	17,185	57.8 (16.6)	57.8	94.2	5.8	0.2	11.1	60.2	28.7
Houston sites									
Intervention									
A	4880	52.6 (13.7)	68.8	8.0	19.7	72.3	83.8	4.8	11.5
I	8527	53.3 (13.7)	70.7	2.7	67.6	29.6	82.9	1.5	15.6
L	5867	51.9 (12.0)	72.6	13.1	9.3	77.6	94.5	0.8	4.7
Total	19,274	51.0 (13.0)	72.0	6.0	94.0	67.0	86.7	2.1	11.2
Control									
P	6388	51.8 (13.4)	73.0	4.1	13.9	82.0	91.7	1.1	7.1
T	5547	50.9 (12.9)	69.5	11.1	28.7	60.2	90.8	2.3	6.9
V	8969	50.7 (13.2)	73.7	4.0	35.6	60.3	95.1	0.6	4.3
Total	20,904	53.0 (13.0)	71.0	7.0	93.0	55.0	92.9	1.2	5.8

^a^Also includes Other/self-pay/indigent/charity care

### Year 1 - RCCT

Table 3 shows the influenza vaccination rates for each site and each intervention group for the baseline and intervention periods. Vaccination rates ranged from a low of 23.6 % to a high of 62 % at baseline across all sites. During the intervention, Pittsburgh intervention sites significantly increased influenza vaccination rates an average of 5.0 PP (*P* < 0.001), while control sites significantly increased influenza vaccination an average of 6.5 PP (*P* < 0.001). In Houston, intervention sites significantly increased influenza vaccination an average 2.7 PP, while control sites increased influenza vaccination an average of 5.2 PP (*P* < 0.001). Influenza vaccination increased more in both the control groups than intervention groups (*P* < 0.001). At the same time, the percent of patients with at least one missed opportunity to vaccinate decreased in the Pittsburgh intervention and control groups and the Houston control group (*P* < 0.001).

**Table 3:** Influenza vaccination rates and missed vaccination opportunities during the Year 1 randomized controlled cluster trial by practice, intervention group and city

Site	Total *N*	% Vaccinated			% of Patients with ≥1 Missed Opportunities		
		Baseline	Year 1	PP Difference	Baseline	Year 1	PP Difference
		8/1/2012-1/31/2013	8/1/2013-1/31/2014		8/1/2012-1/31/2013	8/1/2013-1/31/2014	
Pittsburgh sites							
Intervention							
B	529	49.0	50.5	1.5	79.0	76.0	-3.0
C	2179	57.9	65.0	7.1	65.9	62.6	-3.3
D	3224	54.6	59.3	4.7	66.1	64.1	-2.0
E	1392	47.4	54.2	6.8	78.0	71.2	-6.8
G	417	51.8	54.0	2.2	70.5	69.5	-1.0
H	306	53.3	48.0	-5.3	77.8	69.9	-7.9
F	3611	56.0	60.6	4.6	66.7	67.0	0.3
J	603	49.1	54.7	5.6	70.7	65.7	-5.0
K	330	23.6	30.9	7.3	91.5	89.4	-2.1
M	595	62.0	68.1	6.1	66.9	62.2	-4.7
Total	13,186	53.7	58.7	5.0*^†^	69.3	66.8	-2.5*^†^
Control							
N	2102	60.2	61.8	1.6	71.2	67.4	-3.8
O	4324	35.3	50.9	15.6	76.5	68.9	-7.6
R	2534	42.3	46.7	4.4	76.1	70.5	-5.6
S	1045	35.3	36.0	0.7	83.8	81.3	-2.5
U	2612	52.9	54.2	1.3	68.2	71.2	3.0
W	224	61.2	75.0	13.8	66.1	53.6	-12.5
X	1010	47.2	56.6	9.4	72.5	70.1	-2.4
Y	3334	54.3	58.2	3.9	70.6	68.2	-2.4
Total	17,185	46.8	53.3	6.5*^†^	73.4	69.8	-3.6*^†^
Houston sites							
Intervention							
A	4880	43.4	47.9	4.5	79.3	78.3	-1.0
I	8527	33.2	37.4	4.2	82.7	85.0	2.3
L	5867	36.1	35.2	-0.9	82.0	86.2	4.2
Total	19,274	36.7	39.4	2.7*^†^	81.6	83.7	2.1*^†^
Control							
P	6388	36.6	46.9	10.3	82.5	79.8	-2.7
T	5547	32.2	40.7	8.5	82.6	79.5	-3.1
V	8969	47.0	46.4	-0.6	73.8	76.1	2.3
Total	20,904	39.9	45.1	5.2*^†^	78.8	78.1	-0.7*^†^

*Note*: *PP* percentage point difference between baseline and Year 1 vaccination rates and percent of patients with ≥1 missed opportunities. A decrease in missed opportunities is the desired outcome

^*^ *P* value is a two-sided probability from Cochran-Armitage trend test for percentage point difference between Baseline and Year 1 (*P* < 0.001)

^†^ *P* value is a two-sided probability from chi-square test for percentage point difference between intervention and control groups (*P* < 0.001)

In GEE regressions (Table 4), in the Pittsburgh sites, which had higher baseline rates, likelihood of influenza vaccination was significantly higher for females, older patients, white patients and those with commercial insurance or Medicare. While those in the intervention group were not more likely to receive influenza vaccine, those patients in practices having fewer patients with at least one missed opportunity were more likely to receive the vaccine (*P* < 0.05). In the Houston sites, the likelihood of influenza vaccination was significantly higher among those who were older, of Hispanic ethnicity, in the intervention group and in practices with fewer missed opportunities (*P* < 0.05).

**Table 4:** Likelihood of influenza vaccination at the end of the year 1 randomized controlled cluster trial (1/31/2014) using generalized estimating equations, by city

Variable	Pittsburgh		Houston	
	Odds Ratio (95 % CI)	*P* Value	Odds Ratio (95 % CI)	*P* Value
Patient level variables				
Female, ref. = male	1.08 (1.03-1.13)	0.002	0.98 (0.93-1.03)	0.170
Age, years	1.04 (1.04-1.04)	<0.001	1.03 (1.03-1.03)	<0.001
White race, ref. = Non-white	1.16 (1.04-1.30)	0.007	--	--
Hispanic ethnicity, ref. = Non-Hispanic	--	--	1.12 (1.08-1.16)	<0.001
Medicaid, charity care, uninsured, ref. = Commercial insurance + Medicare	0.97 (0.89-1.05)	0.050	0.95 (0.88-1.02)	0.153
Site level variables				
Intervention, ref. = Control	1.00 (0.95-1.05)	0.950	1.06 (1.00-1.13)	0.048
Patients with ≥1 missed opportunities, %	0.94 (0.94-0.95)	<0.001	0.94 (0.94-0.95)	<0.001

### Year 2 – pre-post study

During the Year 2 pre-post study, vaccination rates (Table 5) among the active intervention groups in the pre-intervention period were 54.5 % for Pittsburgh and 44.7 % for Houston; whereas vaccination rates for the maintenance groups were 55.2 % for Pittsburgh and 40.2 % for Houston, at the beginning of their maintenance period. At the end of the Year 2 intervention period, both the active intervention and maintenance groups significantly increased influenza rates and decreased proportion of patients with missed opportunities in both cities. In bivariate analyses, the change in influenza vaccination did not differ between groups, in Pittsburgh, but was significantly higher in the active intervention group than the maintenance group in Houston (*P* < 0.001)

**Table 5:** Influenza vaccination rates and missed opportunities during the year 2 pre-post study by practice, intervention group and city

Site	Total *N*	% Vaccinated			% Patients with ≥1 Missed Opportunities		
		Pre	Post	PP Difference	Pre	Post	PP Difference
		8/1/2013-1/31/2014	8/1/2014-1/31/2015		8/1/2013-1/31/2014	8/1/2014-1/31/2015	
Pittsburgh sites							
Maintenance							
B	529	50.5	53.3	2.8	76.0	60.9	-15.1
C	2179	65.0	63.4	-1.6	62.6	60.1	-2.5
D	3224	59.3	61.3	2.0	64.1	61.8	-2.3
E	1392	54.2	49.1	-5.1	71.2	73.2	2.0
G	417	54.0	62.1	8.2	69.5	64.3	-5.2
H	306	48.0	50.0	2.0	69.9	73.2	3.3
Total	8047	55.2	56.5	1.4*	68.9	65.6	-3.3*
Active Intervention							
F	3611	60.6	65.3	4.7	67.0	58.7	-8.3
J	603	54.7	54.9	0.2	65.7	65.3	-0.4
K	330	30.9	28.2	-2.7	89.4	92.4	3.0
M	595	68.1	79.5	11.4	62.2	52.6	-9.6
N	2102	61.8	59.0	-2.8	67.4	65.5	-1.9
O	4324	50.9	46.3	-4.6	68.9	68.7	-0.2
R	2534	46.7	41.9	-4.8	70.5	73.5	3.0
S	1045	36.0	40.1	4.1	81.3	81.4	0.1
U	2612	54.2	58.2	4.0	71.2	62.5	-8.7
W	224	75.0	75.9	0.9	53.6	53.6	0
X	1010	56.6	59.9	3.3	70.1	64.0	-6.1
Y	3334	58.2	61.8	3.6	68.2	59.8	-8.4
Total	22,324	54.5	55.9	1.44*	69.6	66.5	-3.1*
Houston sites							
Maintenance							
A	4880	47.9	48.4	0.5	78.3	79.2	0.9
I	8527	37.4	35.3	-2.1	85.0	86.5	1.5
L	5867	35.2	40.0	4.9	86.2	81.6	-4.6
Total	19,274	40.2	41.2	1.7*^†^	83.2	82.4	-0.7**^†^
Active intervention							
P	6388	46.9	49.8	2.9	79.8	75.9	-3.9
T	5547	40.7	46.3	5.7	79.5	73.5	-6.0
V	8969	46.4	48.8	2.4	76.1	74.9	-1.2
Total	20,904	44.7	48.3	3.6*^†^	78.5	74.8	-3.7*^†^

*Note*: *PP* Percentage point difference between pre (Year 1) and post (Year 2). A decrease in missed opportunities is the desired outcome

^*^ *P* value is a two-sided probability from Cochran-Armitage trend test for difference between pre and post intervention (*P* < 0.001)

**P value is a two-sided probability from Cochran-Armitage trend test for difference between pre and post intervention (*P* < 0.05)

^†^ *P* value is a two-sided probability from chi-square test of PP differences between intervention arms

In GEE regression analyses (Table 6) in Pittsburgh, older age, being female, as well as being in the intervention group and being a patient at a site with fewer missed opportunities were related to increased likelihood of influenza vaccination (*P* < 0.05). Similarly in Houston, older age, being female, Hispanic, in the intervention group and in a site with fewer missed opportunities were all related to increased likelihood of influenza vaccination (*P* < 0.01).

**Table 6:** Likelihood of influenza vaccination at the end of the year 2 pre-post study (1/31/2015) using generalized estimating equations, by city

Variable	Pittsburgh		Houston	
	Odds Ratio (95 % CI)	*P* Value	Odds Ratio (95 % CI)	*P* Value
Patient level variables				
Female, ref. = male	1.12 (1.02-1.22)	<0.030	1.12 (1.08-1.16)	<0.001
Age, years	1.04 (1.04-1.04)	<0.001	1.03 (1.02-1.03)	<0.001
White race, ref. = Non-white	0.99 (0.85-1.13)	0.586	--	--
Hispanic ethnicity, ref. = Non-Hispanic	--	--	1.25 (1.21-1.29)	<0.001
Medicaid, charity care, uninsured, ref. = Commercial insurance + Medicare	1.03 (0.91-1.15)	0.856	1.05 (0.95-1.16)	0.357
Site level variables				
Active intervention, ref. = Maintenance group	1.08 (1.03-1.14)	0.003	1.10 (1.40-1.17)	0.002
Patients with ≥1 missed opportunities, %	0.95 (0.95-0.96)	<0.001	0.96 (0.95-0.96)	<0.001

## Discussion

Secular trends in U.S. adult influenza vaccination rates indicate a slow increase in vaccination of approximately 2 percentage points per year. At this rate, it would take nearly 15 years to achieve the national goal of 70 % [4], given the distance between that goal and the current national rate of 42 % [19]. In our randomized controlled cluster trial, all groups increased vaccine uptake by 2.7-6.5 %, suggesting that the intervention using the 4 Pillars™ Practice Transformation Program was not more effective than secular trends. Variation in the level of improvement among practices was noted, with some sites making little to no improvement. Research has shown that impediments to successful quality improvement projects include not having allocated time to devote to the project, lack of leadership support, not having a performance assessment and not having a project champion [20]. In this study, some Champions did not hold positions of influence in the practice and/or were not allowed work time to use the online 4 Pillars™ Program and dashboard to take advantage of the resources and guidance provided therein. Other issues that may have hindered practice change were 1) the lead physician in one small practice was nearing retirement, hence was not engaged in the effort; b) one site was located in a rural Amish community with high vaccine refusal rates; 3) late delivery of influenza vaccine in Year 1 in the Pittsburgh sites; and 4) feedback to the sites on immunization progress was delayed.

When the control groups were offered the intervention and the intervention groups were in maintenance, small additional gains in influenza vaccination rates were realized in the active intervention groups, with no loss among maintenance groups, suggesting that behavior changes to improve vaccination were persisting.

Research has shown that missed opportunities to vaccinate are frequently associated with low vaccination rates [21–24]. In one study, among unvaccinated high risk adults, 90 % reported at least one visit in which influenza vaccine could have been administered [25]. The regression analyses in this study supported those findings and indicated that reducing missed opportunities is a critical element in increasing influenza vaccination. The intervention increased the likelihood of influenza vaccination when missed opportunities decreased in the practices. The 4 Pillars™ Program recommends standing order protocols for clinical staff to offer vaccines, reviewing vaccination status at every visit and offering express vaccine services such as influenza vaccine-only clinics, and walk-in vaccinations during influenza vaccination season. Consistent use of these strategies should reduce the number of missed opportunities and in turn increase vaccination rates [11, 26]. We believe that routine assessment of vaccination status that triggers standing orders is a powerful combination (Pillar 3).

### Strengths and limitations

The strengths of this study are its randomized design, the large number and diversity of patients and practice settings including safety net clinics, and two intervention years of vaccination reporting. These factors enhance its generalizability. The study’s limitations include late delivery of influenza vaccine in Year 1 in the Pittsburgh sites that may have diminished the intervention effect. During the first intervention year, delivery of the EMR data was delayed, preventing the research team from providing feedback about their progress to the sites in both cities early in the intervention. Increases in vaccinations in the control arm when those sites were not in an intervention group may be due to a Hawthorne effect or transference from the intervention and have been reported in other studies [27]; secular trends are smaller than the changes noted in this study.

## Conclusions

As the national adult influenza vaccination rate continues to creep towards national goals, strategies are needed to jumpstart efforts to increase the rate of improvement. An intervention that includes the 4 Pillars™ Practice Transformation Program can assist primary care practices with reducing missed opportunities to vaccinate thus increasing adult influenza vaccination rates.

## Abbreviations

CDC: Centers for Disease Control and Prevention
EMR: Electronic medical record
FM: Family medicine
FM PittNet: Family Medicine Research Network Pittsburgh
GEE: Generalized estimating equation
IM: Internal medicine
PBRN: Practice-based research network
RCCT: Randomized controlled cluster trial
SPUR-Net: Southern Primary-care Urban Research Network Houston

## References

[1] Flu Vaccination Coverage, United States, 2014-15 Influenza Season [http://www.cdc.gov/flu/fluvaxview/coverage-1415estimates.htm] Accessed 27 May 2016.

[2] FluVax View [http://www.cdc.gov/flu/fluvaxview/index.htm] Accessed 27 May 2016.

[3] Influenza (Flu): Influenza vaccination coverage estimates by State, HHS Region, and the United States, National Immunization Survey (NIS) and Behavioral Risk Factor Surveillance System (BRFSS), 2009-10 influenza season [http://www.cdc.gov/flu/fluvaxview/reportshtml/reporti0910/reportii/index.html] Accessed 5 May 2016.

[4] Healthy People 2020: Immunization and Infectious Diseases Objectives [http://www.healthypeople.gov/2020/topics-objectives/topic/immunization-and-infectious-diseases/objectives] Accessed 5 May 2016.

[5] Jessop AB, Dumas H, Moser CA (2013). Delivering influenza vaccine to high-risk adults: subspecialty physician practices. Am J Med Qual.

[6] Nowak GJ, Sheedy K, Bursey K, Smith TM, Basket M (2015). Promoting influenza vaccination: Insights from a qualitative meta-analysis of 14 years of influenza-related communications research by U.S. Centers for Disease Control and Prevention (CDC). Vaccine.

[7] Seasonal Influenza Vaccine Supply and Distribution in the United States [http://www.cdc.gov/flu/about/qa/vaxdistribution.htm] Accessed 14 June 2016.

[8] Koh HK, Sebelius KG (2010). Promoting prevention through the affordable care act. New Engl J Med.

[9] Stewart AM, Cox MA (2013). State law and influenza vaccination of health care personnel. Vaccine.

[10] Influenza Vaccination Honor Roll [http://www.immunize.org/honor-roll/influenza-mandates/honorees.asp] Accessed 27 May 2016.

[11] Guide to Community Preventive Services [https://www.thecommunityguide.org/sites/default/files/assets/What-Works-Vaccines-factsheet-and-insert.pdf]. Accessed 6 May 2016.

[12] Zimmerman RK, Brown AE, Pavlik VN, Moehling KK, Raviotta JM, Lin CJ, Zhang S, Hawk M, Kyle S, Patel S et al. Using the 4 Pillars™ Practice Transformation Program to increase pneumococcal immunizations for older adults: A cluster randomized trial. J Am Geriatr Soc. 2016 (In Press). 10.1111/jgs.14451 PMC5258838 27755655

[13] Campbell MK, Piaggio G, Elbourne DR DGA (2012). Consort 2010 statement: extension to cluster randomised trials. BMJ.

[14] Melinkovich P, Hammer A, Staudenmaier A, Berg M (2007). Improving pediatric immunization rates in a safety-net delivery system. Jt Comm J Qual Patient Saf.

[15] Baskerville NB, Liddy C, Hogg W (2012). Systematic review and meta-analysis of practice facilitation within primary care settings. Ann Fam Med.

[16] Fixsen D, Naoom S, Blase K, Friedman R, Wallace F (2005). Implementation Research: A Synthesis of the Literature.

[17] Saltelli A, Tarantola S, Campolongo F, Ratto M (2004). Sensitivity Analysis in Practice: A Guide to Assessing Scientific Models.

[18] Agresti A (2002). Categorical Data Analysis.

[19] Flu Vaccination Coverage, United States, 2013-14 Influenza Season [http://www.cdc.gov/flu/fluvaxview/coverage-1314estimates.htm] Accessed 14 June 2016.

[20] Watts B, Lawrence RH, Singh S, Wagner C, Augustine S, Singh MK (2014). Implementation of quality improvement skills by primary care teams: case study of a large academic practice. J Prim Care Community Health.

[21] Fu LY, Weissman M, McLaren R, Thomas C, Campbell J, Mbafor J, Doshi U, Cora-Bramble D (2012). Improving the quality of immunization delivery to an at-risk population: a comprehensive approach. Pediatrics.

[22] Nowalk MP, Zimmerman RK, Feghali J (2004). Missed opportunities for adult immunization in diverse primary care office settings. Vaccine.

[23] Nowalk MP, Zimmerman RK, Cleary SM, Bruehlman RD (2005). Missed opportunities to vaccinate older adults in primary care. J Am Board Fam Pract.

[24] Djibo DA, Peddecord KM, Wang W, Ralston K, Sawyer MH (2015). Factors associated with missed opportunities for influenza vaccination review of medical records in a diverse sample of primary care clinics, San Diego County, 2010-2011. J Prim Care Community Health.

[25] Lu P-j, O'Halloran A, Ding H, Srivastav A, Williams WW (2016). Uptake of influenza vaccination and missed opportunities among adults with high-risk conditions, United States, 2013. Am J Med.

[26] Stone EG, Morton SC, Hulscher ME, Maglione MA, Roth EA, Grimshaw JM, Mittman BS, Rubenstein LV, Rubenstein LZ, Shekelle PG (2002). Interventions that increase use of adult immunization and cancer screening services: a meta-analysis. Ann Intern Med.

[27] Pavlik VN, Chan W, Hyman DJ, Feldman P, Ogedegbe G, Schwartz JE, McDonald M, Einhorn P, Tobin JN (2015). Designing and evaluating health systems level hypertension control interventions for African-Americans: lessons from a pooled analysis of three cluster randomized trials. Curr Hypertens Rev.

